# Decapneization as supportive therapy for the treatment of status asthmaticus: a case report

**DOI:** 10.1186/s13256-021-02689-6

**Published:** 2021-04-08

**Authors:** Rossella Esposito, Irene Esposito, Francesco Imperatore, Giovanni Liguori, Fabrizio Gritti, Chiara Cafora, Paolo Francesco Marsilia, Maria De Cristofaro

**Affiliations:** 1 Unit of Intensive Care, Department of Emergency, “A. Cardarelli” Hospital, Naples, Italy; 2 AORN Cardarelli: Azienda Ospedaliera di Rilievo Nazionale Antonio Cardarelli, Naples, Italy

**Keywords:** Asthma, Decapneization, Protective mechanical ventilation, Dynamic hyperinflation

## Abstract

**Background:**

Acute severe asthma is a life-threatening medical emergency. Characteristics of asthma include increased airway resistance and dynamic pulmonary hyperinflation that can manifest in dangerous levels of hypercapnia and acidosis, with significant mortality and morbidity. Severe respiratory distress can lead to endotracheal intubation followed by mechanical ventilation, which can cause increased air trapping with dynamic hyperinflation, predisposing the lungs to barotraumas.

**Case presentation:**

The present case report describes the use of the minimally invasive ECCO_2_R ProLUNG^®^ (Estor) with protective low-tidal-volume ventilation, in a Caucasian patient with near-fatal asthma and with no response to conventional therapy.

**Conclusions:**

Since hypercarbia rather than hypoxemia is the primary abnormality in status asthmaticus, a rescue therapeutic strategy combining the ECCO_2_R membrane ProLUNG^®^ (Estor) with ultra-protective low-tidal-volume ventilation can be successfully applied to limit the risk of severe barotrauma during invasive mechanical ventilation. ECCO_2_R ProLUNG^®^ is a partial respiratory support technique that, based on the use of an extracorporeal circuit with a gas-exchange membrane, achieves relevant CO_2_ clearance directly from the blood using double-lumen venous-venous vascular access, at blood flow in the range of 0.4–1.0 L/minute.

## Background

Asthma is a heterogeneous disease, usually characterized by chronic airway inflammation [1]. It is defined by a history of respiratory symptoms such as wheeze, shortness of breath, chest tightness, and cough that vary over time and in intensity, together with variable expiratory airflow limitation. [2] 3 Characteristics of asthma include increased airway resistance and dynamic pulmonary hyperinflation that lead to increased work of breathing, ventilation–perfusion mismatch, and adverse cardiopulmonary interactions.

Status asthmaticus is difficult to define, but we can affirm that any patient not responding to initial doses of nebulized bronchodilators should be considered to have status asthmaticus [4]. Risk factors for fatal asthma are underestimation of severity and undertreatment. For this reason, once severe respiratory distress is observed, endotracheal intubation should be performed promptly. Mechanical ventilation poses a very difficult challenge because it can easily worsen the status of dynamic hyperinflation, with consequences on the hemodynamic asset. The guidelines on mechanical ventilation for asthma suggest using a protective strategy. It consists of low tidal volume of 5–6 mL/kg, a respiratory rate of 10–12 breaths/minute, and a short inspiratory time to obtain a longer expiratory time (≥ 4 seconds).

This setting can easily lead to a further increase in CO_2_ and thus to hypercapnic acidosis. Therefore, a balance is required between the hypoventilation aiming to protect the lung from ventilator-induced lung injury and the gas exchange. For this purpose, a successful strategy may be to combine a decapneization technique with our protective ventilation, which otherwise could not be applied because of hypercapnia.

In this way, we can remove the excess CO_2_ through the ProLUNG^®^ (Estor) with no modifications of ventilator settings. A CO_2_ removal machine allows for rescue time to treat the lung illness. Decapneization represents an intermediate level between pulmonary ventilation and extracorporeal membrane oxygenation (ECMO). It is a simple veno-venous system with low blood flow that, thanks to the presence of an oxygenator and a blood filter, enables the removal of 20 to 35% of carbon dioxide. Blood is taken, treated, and then reinfused via a single double-lumen catheter inserted into the femoral or jugular vein. A membrane oxygenator for CO_2_ removal is used alone or in combination with continuous renal replacement therapy (RRT). In our case, CO_2_ removal was used alone. It is not a method for oxygenation, so we cannot use it as “lung replacement” therapy, but rather as “lung support” or “protective” therapy.

## Case presentation

**Fig. 1:**
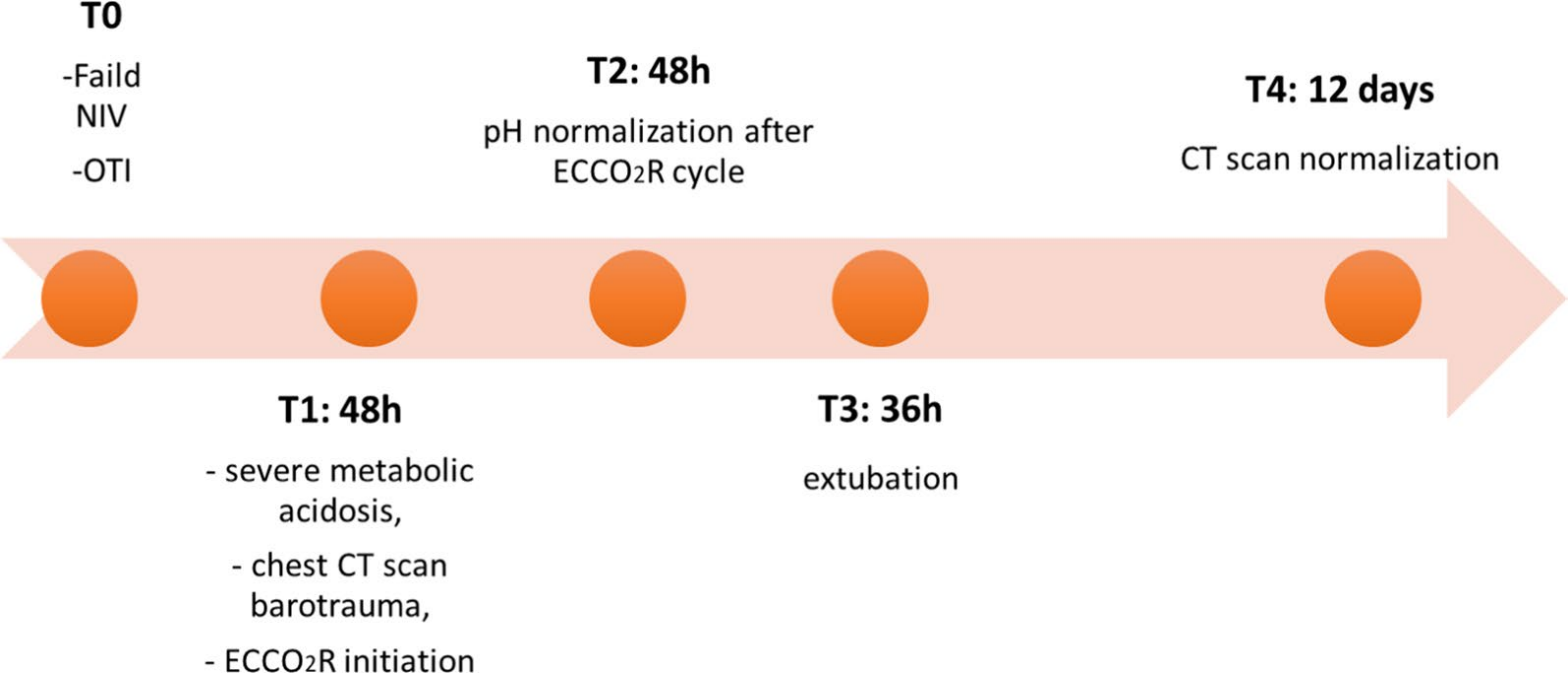
Chronological succession of important events of the patient's hospitalization

**Fig. 2:**
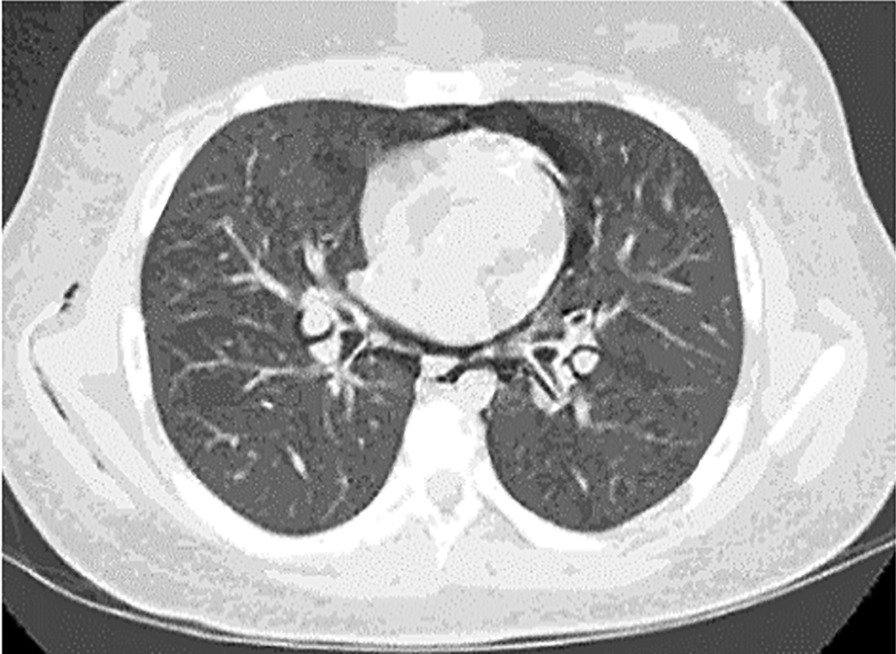
Chest computed tomography scan. Pneumomediastinum with modest aerial elevation which is distributed at the level of the peribronchovascular hilar and perilary spaces on both sides. No pneumothorax was identified

**Fig. 3:**
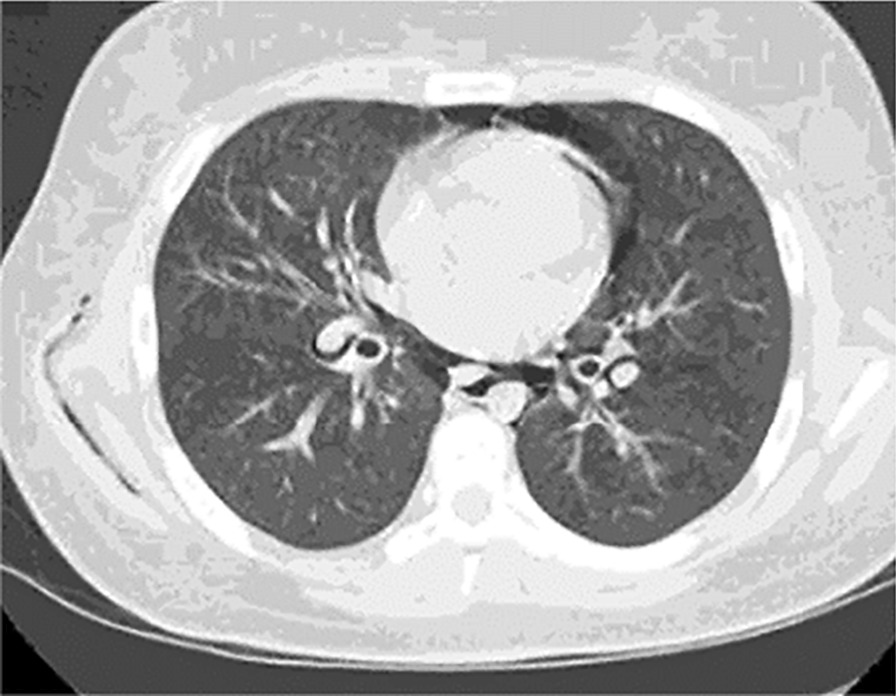
Chest computed tomography scan. Pneumomediastinum with modest aerial elevation which is distributed at the level of the peribronchovascular hilar and perilary spaces on both sides. No pneumothorax was identified

A 16-year-old Caucasian woman with a known history of asthma was admitted to the hospital for severe asthma exacerbation. No significant medical family history or any past severe asthmatic events were reported. The clinical examination showed widespread wheezing and shortness of breath. The initial blood gas showed pH 7.20, partial pressure of oxygen (PaO_2_) of 53 mm Hg, and partial pressure of carbon dioxide (PaCO_2_) of 50 mm Hg. She failed to respond to nebulized salbutamol and ipratropium and to intravenous salbutamol and magnesium, and therefore was transferred to the intensive care unit (ICU), where noninvasive ventilation (NIV) was started. Despite this strategy, she exhibited pronounced use of accessory respiratory muscles and rapidly required orotracheal intubation (OTI). The ventilator was set according to guidelines of mechanical ventilation for severe acute asthma, based on low tidal volume 5–6 mL/kg of ideal body weight (IBW), respiratory rate of 10–12 breaths/minute, and prolonged expiratory time. Sedation and neuromuscular-blocking agents were needed to ensure this degree of hypoventilation. The dynamic hyperinflation was assessed by measuring the plateau pressure (after a transient inspiratory pause), which was about 30 mmHg with low blood pressure. We performed a 60-second apnea test, which confirmed the correlation between the lung hyperinflation and the hemodynamic sufferance. Therefore, the ventilatory patterns were modified by reducing tidal volume to 4 mL/kg and respiratory rate to 10 breaths/min (timeline T0) (Fig. 1). After 48 hours of ventilation, the arterial blood gas showed increased PaCO_2_ (more than 115 mmHg), pH of 7.01, and PaO_2_ of 60 mmHg. A first chest computed tomography (CT) scan was performed (Figs. 2, 3), which revealed pneumomediastinum.

The worsening of alveolar hypoventilation following these ventilator settings, and the extension of the pulmonary barotraumas prompted the implantation of an ECCO_2_R ProLUNG^®^ membrane (Estor) (timeline T1) (Fig. 1). Under ultrasound guidance, a 13.0 French double-lumen catheter was inserted into the right femoral vein using the Seldinger technique. The system was primed with saline and heparin and attached to the vascular catheter. The CO_2_ filtration fraction was 20%. The decapneization period lasted about 48 hours, in conjunction with drug therapy consisting of nebulized adrenaline (2–4 mg in 2–4 mL), 30 mcg/minute of salbutamol by continuous infusion, and 40 mg/day of intravenously administered methylprednisolone. No adjustments were made to the setting of the mechanical ventilation in order to keep the lung-protective strategy by maintaining very low tidal volumes (about 4 mL/kg) and decreased respiratory rate (RR about 10 breaths/minute). Sedation and neuromuscular-blocking agents continued to be applied. No hypotension or other hemodynamic complications occurred during the CO_2_ removal. Several arterial blood gases were taken to monitor the gas exchange, and a gradual improvement of hypercapnia was observed. After 48 hours of decapneization, the patient’s clinical status was significantly improved, with decreased wheezing and return to normal pH value, with a marked reduction in PaCo_2_ (55 mmHg) (timeline T2) (Fig. 1).

**Fig. 4:**
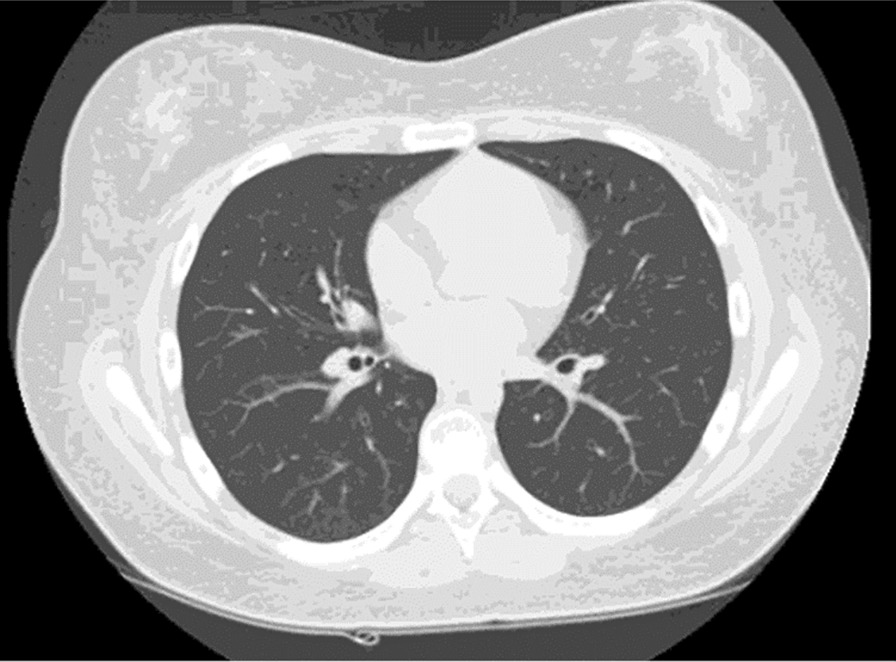
Chest computed tomography (CT) scan. The new chest CT scan shows the disappearance of pneumomediastinum and regular pulmonary expansion

A period of weaning was started, and after 36 hours the patient was extubated and transferred from the ICU to the respiratory care department (timeline T3) (Fig. 1). A second chest CT scan was performed 12 days after the first scan (Fig. 4) and showed the disappearance of barotraumas (timeline T4) (Fig. 1).

## Discussion and conclusions

A substantial percentage (4%) of cases of acute asthma exacerbation require admission to the ICU, and one third of these patients require endotracheal intubation and mechanical ventilation [5, 6].

Risk factors for near-fatal asthma (NFA) requiring invasive mechanical ventilation include a younger age at presentation, poor compliance with drug therapy, poor outpatient follow-up, more than three emergency department visits in the preceding year, recent hospital admission, a prior episode of NFA, and prior mechanical ventilation [7]. In the present case, the patient was poorly compliant with prescribed therapy. The significant heart–lung interaction present in patients with asthma causes hemodynamic instability. An increase in intrathoracic pressure secondary to gas trapping can lead to an acute increase in pulmonary vascular resistance and right heart pressure, with impaired venous return, and then left ventricular diastolic volume and cardiac output. Positive-pressure mechanical ventilation promotes life-threatening complications of status asthmaticus including pneumothorax, pneumomediastinum, giant subcutaneous emphysema, and circulatory shock. Despite the early use of low-tidal-volume (4–6 mL/kg IBW) protective mechanical ventilation in our patient, this strategy was rapidly compromised by severe respiratory acidosis. Plateau pressure (Pplat) was high, as a consequence of worsening bronchospasm with associated gas trapping. In the literature, ECMO is generally recommended in the setting of potentially reversible cardiopulmonary failure. However, although the literature is confined to a few case reports, a recent review of the multicenter international Extracorporeal Life Support Organization registry by Mikkelsen and colleagues [8, 9] revealed that status asthmaticus was the primary indication for ECMO in 24 of 1257 adult patients included in the registry. A total of 20 (83.3%) patients with asthma survived to hospital discharge, whereas only 50.8% of patients with other causes of respiratory failure (odds ratio 4.86) survived therapy. Extracorporeal carbon dioxide removal, when compared to ECMO, is less invasive (requires a single venous catheter and a lower blood flow rate) and limits the risk of barotrauma.

In conclusion, this rescue technique (ECCO_2_R) seems particularly interesting in the case of asthmatic patients where early protective mechanical ventilation (low tidal volume 6 mL/kg IBW) fails. The ECCO_2_R membrane allows the use of ultra-protective mechanical ventilation with a drastic reduction in tidal volume (3–4 mL/kg IBW) and minute ventilation, immediately limiting lung overinflation and gas trapping, and reducing the barotrauma.

## Data Availability

Not applicable; it is a case report.
